# Evolutionary conservation of zinc finger transcription factor binding sites in promoters of genes co-expressed with WT1 in prostate cancer

**DOI:** 10.1186/1471-2164-9-337

**Published:** 2008-07-16

**Authors:** Kurtis Eisermann, Sunpreet Tandon, Anton Bazarov, Adina Brett, Gail Fraizer, Helen Piontkivska

**Affiliations:** 1 School of Biomedical Sciences, Kent State University, Kent, Ohio, USA; 2 Department of Biological Sciences, Kent State University, Kent, Ohio, USA

## Abstract

**Background:**

Gene expression analyses have led to a better understanding of growth control of prostate cancer cells. We and others have identified the presence of several zinc finger transcription factors in the neoplastic prostate, suggesting a potential role for these genes in the regulation of the prostate cancer transcriptome. One of the transcription factors (TFs) identified in the prostate cancer epithelial cells was the Wilms tumor gene (*WT1*). To rapidly identify coordinately expressed prostate cancer growth control genes that may be regulated by WT1, we used an *in silico *approach.

**Results:**

Evolutionary conserved transcription factor binding sites (TFBS) recognized by WT1, EGR1, SP1, SP2, AP2 and GATA1 were identified in the promoters of 24 differentially expressed prostate cancer genes from eight mammalian species. To test the relationship between sequence conservation and function, chromatin of LNCaP prostate cancer and kidney 293 cells were tested for TF binding using chromatin immunoprecipitation (ChIP). Multiple putative TFBS in gene promoters of placental mammals were found to be shared with those in human gene promoters and some were conserved between genomes that diverged about 170 million years ago (i.e., primates and marsupials), therefore implicating these sites as candidate binding sites. Among those genes coordinately expressed with *WT1 *was the kallikrein-related peptidase 3 (*KLK3*) gene commonly known as the prostate specific antigen (*PSA*) gene. This analysis located several potential WT1 TFBS in the *PSA *gene promoter and led to the rapid identification of a novel putative binding site confirmed *in vivo *by ChIP. Conversely for two prostate growth control genes, androgen receptor (*AR*) and vascular endothelial growth factor (*VEGF*), known to be transcriptionally regulated by WT1, regulatory sequence conservation was observed and TF binding *in vivo *was confirmed by ChIP.

**Conclusion:**

Overall, this targeted approach rapidly identified important candidate WT1-binding elements in genes coordinately expressed with WT1 in prostate cancer cells, thus enabling a more focused functional analysis of the most likely target genes in prostate cancer progression. Identifying these genes will help to better understand how gene regulation is altered in these tumor cells.

## Background

In the United States, prostate cancer is the most common form of cancer in men and is the second most deadly cancer in men killing more than 27,000 annually [1]. Nearly one in six men will develop prostate cancer at some point in their life, with the majority of incidences occurring after the age of 50. The major biomarker for prostate cancer diagnosis is prostate specific antigen (PSA), however, the sensitivity and specificity of the PSA assay is limited [2]. Improved biomarkers will result from a better understanding of molecular mechanisms that regulate this disease.

Global gene expression analyses have led to a better understanding of growth control of prostate cancer cells [3-5]. Ongoing studies identified more than 200 genes predominantly expressed in prostate cancer epithelial cells [6] and included genes likely to influence growth of prostate cancer cells, such as growth factors, growth factor receptors and TFs (as identified by Gene Ontology and KEGG pathway analyses). Two of the TFs identified in the prostate cancer epithelial cells were the Wilms tumor gene (*WT1*) and the early growth response gene (*EGR1*), zinc finger transcription factors that bind at G-rich promoters of genes that regulate growth. In fact, the WT1 TF binds at several G-rich sites (GNGNGGGNG), including the EGR1 consensus binding site GCGGGGGCG [7-9]. Both WT1 and EGR1 have been identified in prostate cancer cells, although their function in prostate epithelium is unknown [10-12]. WT1 has an essential role in the normal development of the urogenital system and has been shown to suppress transcription of the promoters of many important growth factors [13].

While identifying prostate growth control pathways potentially regulated by WT1, we have focused our studies on candidate genes belonging to known growth regulatory pathways. We have previously described WT1 regulation of the androgen receptor (*AR*) and vascular endothelial growth factor (*VEGF*) gene promoters [14,15]. To go beyond the candidate genes approach and identify novel gene targets coordinately expressed with WT1 in tumor epithelial cells, a more systematic and unbiased high-throughput computational approach was used. These *in silico *analyses were based on 24 genes expressed in prostate cancer epithelium that were likely to influence growth of prostate cancer cells. Putative TFBS were computationally predicted; however, the identification of functional TFBS is a challenge and requires an alternative approach. Availability of complete genomic sequence from multiple species allows identification of evolutionary conserved elements, e.g. cis-regulatory elements. Functionally important elements are likely to experience purifying selection pressure [16-20], thus, we can utilize the degree of evolutionary conservation to identify TFBS that are likely to be functional. Our approach was to identify regions (and TFBS) evolutionary conserved across multiple mammalian genomes, including those separated by 170 million years (human and opossum) [21]. Overall, this targeted approach identified important candidate binding elements in genes coordinately expressed with WT1 in prostate cancer epithelial cells. Identifying genes regulated by zinc finger TFs expressed in prostate cancer cells will enhance understanding of the altered pathways in these tumor cells and provide useful biomarkers for prostate cancer progression.

## Results

### Evolutionary conservation analysis: TFBS conserved in prostate cancer growth genes

Genomic sequences of proximal promoter regions of 24 genes expressed in prostate cancer epithelial cells (Additional file 1) were analyzed to determine the degree of evolutionary conservation and to identify potentially important regulatory regions. Binding sites for six TFs (WT1, EGR1, SP1, SP2, AP2, and GATA1) were investigated for evolutionary conservation over a range of eight different mammalian species (human, chimpanzee, macaque, cow, dog, mouse, rat and opossum) (Table 1). Tables 2 and 3 highlight 11 of these genes whose promoter sequences could be aligned in at least five mammalian species (human, chimpanzee, macaque, rat and mouse) and were found to have at least one evolutionary conserved TFBS.

**Table 1:** Transcription factors potentially involved in coordinate gene expression in prostate cancer epithelial cells

**Symbol**	**Name**	**Expression in prostate**
WT1	Wilms tumor 1	[10,84]
EGR1	Early growth response 1	[11,12]
SP1	Specificity protein 1	[24,85]
SP2	Specificity protein 2	[86,87]
AP2	Transcription factor AP-2	[88,89]
GATA1	GATA binding protein 1	NR^a^

^a ^None reported.

**Table 2:** Genes co-expressed with WT1 in prostate cancer epithelium ^a^

**Gene symbol**	**Gene name**	**Synonyms**	**Ensembl gene ID**	**Entrez gene ID**	**Summary of function ^b^**	**Regulation ^c,d^**	**Binding ^c,e^**	**Expression in prostate**
*ECAD*	cadherin 1, type 1, E-cadherin (epithelial)	*CDH1*	ENSG00000039068	999	signaling		+	[90]
*EGR1*	early growth response 1	*TIS8, GOS30, AT225*	ENSG00000120738	1958	TF	+, ++	+	[11,12]
*GATA2*	GATA binding protein 2	*NFE1B*	ENSG00000179348	2624	TF	+, ++	+	[56]
*IGFBP2*	insulin-like growth factor binding protein-2	*IBP2*	ENSG00000115457	3485	signaling	+	+, ++	[91-93]
*KLK3*	kallikrein 3, (prostate specific antigen)	*PSA*	ENSG00000142515	354	enzyme, signaling		++	[48,49,94]
*NDRG1*	N-myc downstream regulated gene 1	*DRG1, RTP, TDD5, NDR1*	ENSG00000104419	10397	enzyme		++	[63,95,96]
*NPY*	neuropeptide Y	*PYY4*	ENSG00000122585	4852	signaling		+	[97,98]
*SOX4*	SRY (sex determining region Y)-box-4		ENSG00000124766	6659	TF	+, ++	+	[99,100]
*SOX9*	SRY (sex determining region Y)-box-9	*CMD1, CMPD1, SRA1*	ENSG00000125398	6662	TF	+, ++	+	[101,102]
*TFAP2C*	transcription factor AP-2 gamma	*ERF1, TFAP2G*	ENSG00000087510	7022	TF	+, ++	+	[89]
*WT1*	Wilms tumor 1	*WAGR, WIT-2, GUD*	ENSG00000184937	7490	TF	+, ++	+	[10,84]

^a ^Genes expressed in prostate cancer epithelial cells [6] include those listed in additional file 1.

^b ^Function as defined in the respective SwissProt annotation [103].

^c ^Broad functional categories are based on Gene Ontology [104] functional annotation by DAVID [105].

^d ^+ designates GOTERM_BP_ALL:regulation of biological process, ++ designates GOTERM_BP_ALL:regulation of transcription, GOTERM_BP_ALL:regulation of metabolism.

^e ^+ designates GOTERM_MF_ALL:binding, ++ designates GOTERM_MF_ALL:protein binding.

**Table 3:** TFBS in promoters of genes expressed in prostate cancer are conserved between Human and Primates or Rodents

**Gene**		**WT1**		**EGR1**		**SP1**		**SP2**		**AP2**		**GATA1**	
	
	**Conserved between**^a^	**#Cons. sites**^b^	**Total#**^c^	**#Cons. sites**^b^	**Total# ^c^**	**#Cons. sites**^b^	**Total#**^c^	**#Cons. sites**^b^	**Total#**^c^	**#Cons. sites**^b^	**Total#**^c^	**#Cons. sites**^b^	**Total#**^c^
***ECAD***	**H-Pr**	1/PA	2	1/0	1	3/0	4	1/0	2	1/PA	1	NP/NP	NP
	**H-Ro**	0/0		PA/PA		0/0		0/0		0/0		NP/NP	
***EGR1***	**H-Pr**	4/4	8	7/8	10	3/4	7	1/0	1	1/1	1	3/1	3
	**H-Ro**	2/2		3/4		2/2		0/0		0/0		1/0	
***GATA2***	**H-Pr**	7/8	8	1/1	1	3/4	5	3/2	3	2/3	3	NP/NP	NP
	**H-Ro**	1/2		PA/PA		1/0		0/0		0/0		NP/NP	
***IGFBP2***	**H-Pr**	3/2	3	6/6	6	6/3	7	1/1	1	NP/NP	NP	3/1	3
	**H-Ro**	0/0		3/1		1/1		0/0		NP/NP		0/0	
***KLK3***	**H-Pr**	2/2	3	1/1	1	1/1	2	1/0	1	NP/NP	NP	1/1	2
	**H-Ro**	NSA/NSA		NSA/NSA		NSA/NSA		NSA/NSA		NSA/NSA		NSA/NSA	
***NDRG1***	**H-Pr**	1/1	1	NP/NP	NP	NP/NP	NP	NP/NP	NP	0/1	1	1/1	1
	**H-Ro**	0/0		NP/NP		NP/NP		NP/NP		0/0		0/0	
***NPY***	**H-Pr**	9/7	9	2/1	2	4/4	4	2/2	2	4/2	5	2/2	2
	**H-Ro**	1/1		0/0		1/1		1/0		0/0		0/0	
***SOX4***	**H-Pr**	2/1	2	2/2	2	3/3	3	1/1	1	NP/NP	NP	2/2	2
	**H-Ro**	1/1		0/0		2/2		0/0		NP/NP		2/2	
***SOX9***	**H-Pr**	3/2	4	1/1	1	4/4	4	NP/NP	NP	NP/NP	NP	NP/NP	NP
	**H-Ro**	0/1		0/PA		1/NA		NP/NP		NP/NP		NP/NP	
***TFAP2C***	**H-Pr**	NP/NP	NP	4/4	4	5/4	8	NP/NP	NP	1/1	1	NP/NP	NP
	**H-Ro**	NP/NP		3/3		2/2		NP/NP		1/1		NP/NP	
***WT1***	**H-Pr**	4/2	7	3/3	3	3/3	6	1/1	1	2/1	4	1/0	5
	**H-Ro**	1/1		0/0		2/2		0/0		1/0		0/0	

^a ^H-Pr = TFBS conserved between human and other primates (chimpanzee/macaque), H-Ro = TFBS conserved between human and rodents (mouse/rat).

^b ^Number of conserved sites: PA = only partial alignment of promoters as constructed by MultiPipMaker [79], NP = no TFBS in human promoters as predicted by the MatIspector [75], 0 = TFBS not conserved, and NSA = no orthologous sequence is available in Ensembl.

^c ^Total number of predicted sites is based on TFBS in human promoters.

Among the TFBS investigated, WT1, EGR1 and SP1 sites showed the highest frequency of evolutionary conservation in the gene promoters surveyed. For example, the promoters of *EGR1*, *GATA2 *and *WT1 *were found to have multiple WT1, EGR1 and SP1 candidate binding sites that were conserved through multiple species (Table 3). In the *EGR1 *promoter, 50% of WT1 sites are conserved between human and primates. Additionally, in the *GATA2 *gene promoter, 94% of WT1 sites, 70% of SP1 sites, and 100% of EGR1 sites are conserved between human and other primates (Table 3). Similarly, in the *WT1 *gene promoter 50% of SP1, 43% of WT1 and 100% of EGR1 sites are conserved between human and other primates (Table 3). WT1, EGR1, and SP1 TFBS within the promoters of *IGFBP2, KLK3, NPY, SOX4, SOX9*, and *TFAP2C *are also conserved between human and other primates (Table 3).

Importantly, for the *WT1 *and *EGR1 *gene promoters this conservation extended into the marsupials (Table 4). The *EGR1 *gene promoter is relatively conserved between human and opossum with 20% of predicted EGR1, 12% of predicted WT1 and 14% of predicted SP1 sites conserved between human and opossum. Similarly, the *WT1 *gene promoter exhibited conservation between human and opossum, with 33% of predicted SP1 and 14% of predicted WT1 sites shared between human and opossum. In the *GATA2 *promoter only 12% of predicted WT1 sites are shared between human and opossum (Table 4). Overall, TFBS for the three TF (WT1, EGR1, and SP1) were evolutionary conserved between human and the distantly related species, opossum, in seven different promoters (*WT1, EGR1, GATA2, IGFBP2, SOX4, SOX9, and TFAP2C*).

**Table 4:** TFBS in promoters of genes expressed in prostate cancer are conserved between Human and Opossum

**Gene**		**WT1**		**EGR1**		**SP1**		**AP2**	
	
	**Conserved between**^a^	**#Cons. sites**^b^	**Total#**^c^	**#Cons. sites**^b^	**Total#**^c^	**#Cons. sites**^b^	**Total#**^c^	**#Cons. sites**^b^	**Total#**^c^
***EGR1***	H-Op	1	8	2	10	1	7	PA	1
***GATA2***	H-Op	1	8	0	1	PA	5	0	3
***IGFBP2***	H-Op	0	3	1	6	1	7	NP	NP
***NPY***	H-Op	0	9	0	2	0	4	1	5
***SOX4***	H-Op	0	2	2	2	0	3	NP	NP
***TFAP2C***	H-Op	NP	NP	1	4	2	8	PA	1
***WT1***	H-Op	1	7	0	3	2	6	PA	4

^a ^H-Op = Conservation between human and opossum.

^b ^Number of conserved sites: PA = only partial alignment of promoters as constructed by MultiPipMaker [79], NP = no TFBS in human promoters as predicted by the MatIspector [75], and 0 = TFBS not conserved.

^c ^Total number of predicted sites is based on sites in human promoters.

Tables 3 and 4 show that there were fewer SP2, AP2 and GATA1 than WT1, EGR1 and SP1 TFBS in the 11 gene promoters analyzed. While evolutionary conservation between primates was similar for all six TFBS, conservation between human and rodents diminished for SP2 and AP2 TFBS. AP2 sites in the promoters of the *GATA2*, *WT1*, and *NPY *genes showed 25% to 100% conservation between human and other primates. Conservation of AP2 sites was the strongest in the *NPY *gene promoter as these sites are also conserved between human and opossum (Table 4). In addition to conservation of GC-rich TFBS, the AT-rich GATA1 binding sites were shown to be highly conserved in several gene promoters including *SOX4, EGR1, IGFBP2 *and *NPY *(Table 3). All of the GATA1 sites in these four promoters are conserved between human and chimpanzee, and for the *SOX4 *gene promoter this strong conservation extends to rodents as well.

The overall evolutionary conservation of predicted TFBS of these 11 different genes expressed in prostate cancer cells was analyzed. As would be expected, conservation of TFBS decreased as species became more evolutionarily divergent (Table 5). TFBS were found to be the most conserved among primates, followed by rodents, and the least amount of conservation was found between human and opossum. Of the 47 predicted WT1 sites in the 11 genes analyzed, primates had 68% of these sites conserved between human and primate genomes, while rodent genomes had only 15% of these sites being conserved, and opossum only 6% of these sites conserved, clearly showing a drastic drop in conservation as species diverge. This same pattern was shown for the other TFBS that were analyzed, including EGR1 and SP1. In particular, 85% of the EGR1 sites were conserved between human and other primates, 26% between human and rodents, and 19% between human and opossum. Similarly, there were 50 predicted SP1 binding sites, of which 62%, 22% and 12% were conserved between human and primates, rodents, and opossum genomes, respectively, therefore, exhibiting decreasing conservation of these sites with evolutionary divergence. Thus, with this approach of identifying evolutionary conserved sequences we were able to pinpoint specific candidate binding sites that could be tested for functional relevance.

**Table 5:** Summary of evolutionary conserved sites shared between genomes of human and other species

	**WT1**	**EGR1**	**SP1**	**SP2**	**AP2**	**GATA1**
**Total # of TFBS^a^**	**47**	**31**	**50**	**12**	**16**	**18**
						
**Primates**						
Chimpanzee	36 (77%)	28 (90%)	35 (70%)	11 (92%)	11 (69%)	13 (72%)
Macaque	28 (60%)	25 (81%)	27 (54%)	6 (50%)	9 (56%)	8 (44%)
						
**Primate % conserved^b^**	**68%**	**85%**	**62%**	**71%**	**63%**	**58%**
						
**Rodents**						
Mouse	6 (13%)	9 (29%)	12 (24%)	1 (8%)	2 (13%)	3 (17%)
Rat	8 (17%)	8 (26%)	10 (20%)	0 (0%)	1 (6%)	2 (11%)
						
**Rodent % conserved^c^**	**15%**	**26%**	**22%**	**4%**	**9%**	**14%**
						
**Marsupials**						
Opossum	3	6	6	0	1	0
**% conserved**	**6%**	**19%**	**12%**	**0%**	**6%**	**0%**

^a ^Total # of TFBS = The total number of TFBS predicted by MatIspector [75] based on TFBS in human promoters of 11 genes. Numbers of evolutionary conserved TFBS shared between human and each species are shown below (percent of sites conserved shown in parenthesis).

^b ^Primate % conserved = Average number of chimpanzee and macaque evolutionary conserved TFBS divided by the total number of TFBS predicted for that particular TF.

^c ^Rodent % conserved = Average number of mouse and rat evolutionary conserved TFBS divided by the total number of TFBS for that particular TF.

### Conservation of overlapping WT1, EGR1, and SP1 TFBS

**Figure 1:**
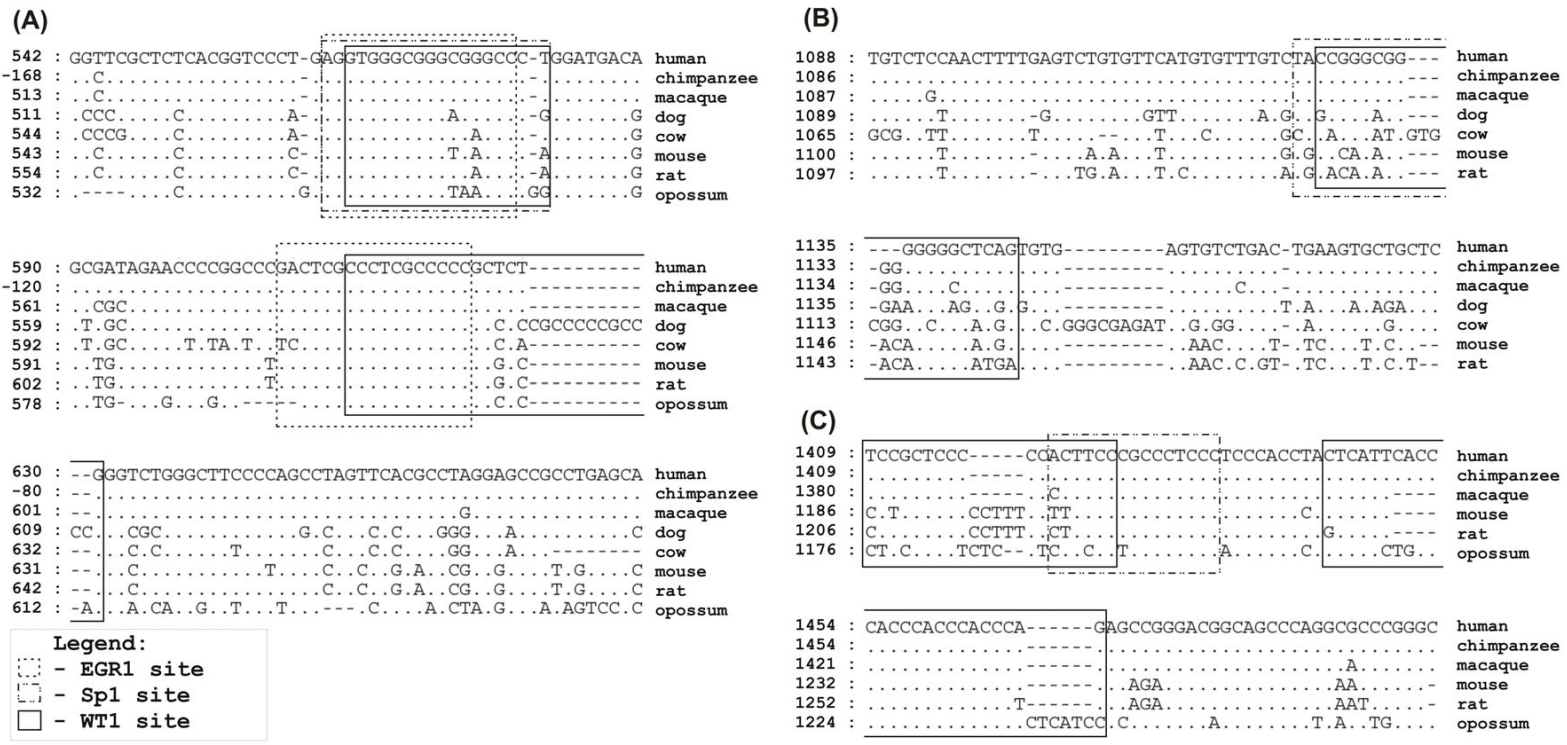
**Alignment of TFBS in *EGR1*, *GATA2*, and *WT1 *promoters reveals overlapping SP1, EGR1 and WT1 sites**. Dots indicate nucleotides identical to human, while gaps are shown with dashes. Predicted TFBS are based on human sequences and are marked by boxes: EGR1, dashed; SP1, dash-dotted; WT1, solid. **(A) **Two separate WT1 sites in the *EGR1 *promoter are conserved between multiple species and both overlap an EGR1 site, and one also overlaps an SP1 site. WT1 site (human 614–630) overlaps EGR1 site (human 608–624) and both sites are conserved between all eight species surveyed. The WT1 site (human 565–581) overlaps both an EGR1 site (human 563–575) and an SP1 site (human 563–577). The SP1 site is conserved between all eight species, the WT1 site is conserved between all but opossum and the EGR1 site is conserved between primates. Negative numbers in the chimpanzee *EGR1 *promoter sequence indicate that the orthologous region was located 1,668 base pairs from the ATG site (further upstream than 1.5 kb analyzed for other species). **(B) **Two overlapping WT1 sites (human 1127–1143 and human 1129–1145) overlap an SP1 site (human 1125–1139) in the *GATA2 *gene promoter region. The WT1 sites are conserved between human, chimpanzee, and macaque, while the SP1 site is conserved between human, chimpanzee, macaque, and cow. **(C) **Two WT1 and an SP1 TFBS in the *WT1 *promoter are conserved. The WT1 site (human 1444–1468) is conserved between human, chimpanzee, macaque, mouse, rat, and opossum. The WT1 site (human 1409–1425) that overlaps an SP1 site is conserved between human and chimpanzee only, while the SP1 site (human 1420–1434) is conserved between human, chimpanzee, macaque, mouse, and rat.

Several of the genes investigated have multiple overlapping WT1, EGR1, and SP1 binding sites in their proximal promoter regions. For example, the promoter of the human *EGR1 *gene has evolutionary conserved overlapping WT1/SP1 binding sites (one of which is shown in Figure 1A). Both the overlapping WT1 (human 565–581) and SP1 (human 563–577) sites are conserved between seven of eight species compared, and the SP1 site is also conserved between human and opossum. A second WT1 site (human 614–630) located 33 bp downstream overlaps an EGR1 site (human 608–624) and both sites are conserved among all eight species, including opossum (Figure 1A). The promoter of the *GATA2 *gene also contained overlapping SP1 and WT1 TFBS (located in human positions 1125–1139 and 1127–1145, respectively) that are conserved among several mammalian genomes (Figure 1B). The *WT1 *gene promoter also has overlapping WT1/SP1 binding sites and when aligned with multiple species, one 3' WT1 site (human 1444–1468) was conserved between all primates, rodents, and opossum, thus, depicting millions of years of conservation of this particular site (Figure 1C). The SP1 site (human 1420–1434) is conserved between all primates and rodents tested, and overlaps with a WT1 site (human 1409–1425) that is conserved between human and chimpanzee (Figure 1C). Interestingly, the sequence similarity is so great between human and chimpanzee for this *WT1 *promoter region that no insertions or deletions were observed in either genomic sequence; thus, these TFBS were located in exactly the same positions relative to the start ATG codon.

Identification of overlapping TFBS in the gene promoters indicated that WT1 and EGR1 may compete for binding. Analyses of the promoter regions of 11 genes expressed in prostate cancer epithelial cells showed that WT1 TFBS overlapped SP1 and EGR1 TFBS, either separately or together. Overall, it was found that there were 25 overlapping sites in the promoter regions of these genes. There were 12 WT1/SP1, seven SP1/EGR1, three WT1/EGR1, and three WT1/SP1/EGR1 overlapping sites (Table 6). These overlapping sites were found in 10 of the 11 gene promoters analyzed. Seven overlapping sites were identified in the promoter region of the *EGR1 *gene, and three of these seven overlapping sites are conserved between human and other species. Three other gene promoters, *GATA2, IGFBP2*, and *TFAP2C*, have three overlapping sites each, with one SP1/EGR1 site conserved between human and opossum for both the *TFAP2C *and *IGFBP2 *promoters. The *WT1 *and *KLK3 *promoters have overlapping WT1/SP1 and SP1/EGR1 sites, respectively. All of these overlapping TFBS are excellent candidates for functional testing to determine whether competition for TF binding at these sites results in activation or suppression of the genes they are regulating.

**Table 6:** Conservation of overlapping TFBS between human and other mammals^a^

**Gene**	**WT1/EGR1**	**SP1/EGR1**	**WT1/SP1**	**WT1/SP1/EGR1**
*ECAD*	0	0	1233–1249: C	0
*EGR1*	608–630: Pr, Ro, Op	703–727: Pr	1340–1360: M	563–581: Pr
	1029–1047: C		1466–1486: M	1466–1490: M
*GATA2*	0	0	282–300: M	0
			892–910: Pr	
			1125–1145: Pr	
*IGFBP2*	0	1327–1359: Pr, Ro, Op	716–744: C	0
			1445–1463: C	
*KLK3*	0	1400–1418: Pr	0	0
*NDRG1*	0	0	0	0
*NPY*	512–530: Pr	0	260–278: Pr	0
*SOX4*	0	1344–1362: C	0	0
*SOX9*	0	0	908–932: Pr	908–932: Pr
*TFAP2C*	0	1127–1145: Pr, Ro	0	0
		1160–1179: Pr		
		1384–1403: Pr, Ro, Op		
*WT1*	0	0	1089–1108: C	0
			1409–1434: C	

^a ^Position numbers are based on predicted TFBS in the human sequences. Pr = Both primates (chimpanzee and macaque), C = chimpanzee, M = macaque, Ro = Both rodents (mouse and rat), Op = opossum. 0 = No overlapping WT1/EGR1, SP1/EGR1, WT1/SP1, or WT1/SP1/EGR1 TFBS for that particular gene.

### Sequence conservation of TFBS indicates a potentially functional WT1 binding site in the *KLK3 *(*PSA*) promoter

**Figure 2:**
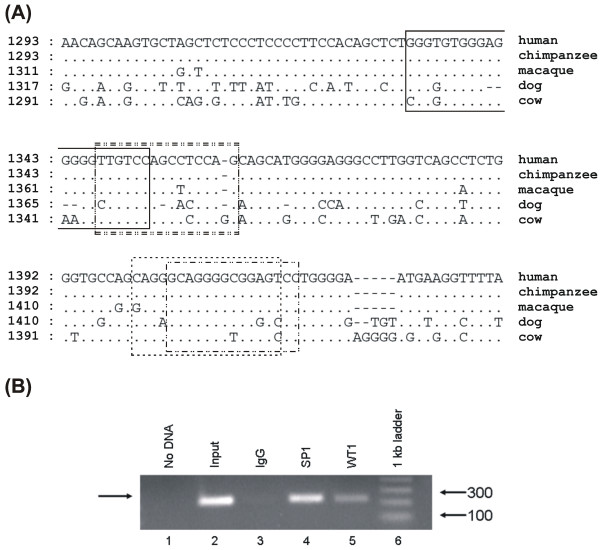
**Conservation of the *KLK3 *(*PSA*) promoter and ChIP verification of WT1 and SP1 binding**. **(A) **Alignment of predicted TFBS (based on human sequences) in the *KLK3 *gene promoter of multiple genomes shows the conservation of two overlapping WT1 binding sites (solid box), an EGR1 site (dashed box), an SP1 site (dash-dotted box), and an SP2 site (double dash-dotted box). WT1 sites (human 1332–1348 and 1336–1352) are conserved between human, chimpanzee, macaque, and cow and they overlap an SP2 site (human 1347–1361) conserved between human, chimpanzee, and cow. An EGR1 site (human 1400–1416) overlaps an SP1 site (human 1404–1418) and both are conserved between human, chimpanzee, macaque, and dog. **(B) **The binding of WT1 and SP1 TFs to native chromatin obtained from WT1-transfected LNCaP cells was confirmed by ChIP. Lane 1 shows the no DNA PCR control and lane 2 shows PCR amplified input DNA. Lanes 3, 4, and 5 show PCR amplified DNA immunoprecipitated by IgG (no antibody control), SP1 or WT1 antibodies, respectively.

One of the 24 genes differentially expressed in prostate cancer epithelial cells was *KLK3 *(*PSA*), an important diagnostic marker. Sequence alignment of the *KLK3 *promoter revealed three WT1 sites and two SP1 sites, with two-thirds of the WT1 and one-half of the SP1 sites conserved between human and other primates (Table 3). Given the premise that evolutionary conserved sites are more likely to be functionally relevant, we tested these conserved sites for their ability to bind TF *in vivo*. PCR primers were designed to flank the region where adjacent conserved WT1 (human 1332–1352) and the SP1 sites (human 1404–1418) were identified (Figure 2A). Both of these binding sites in the *PSA *promoter were tested by chromatin immunoprecipitation (ChIP) in hormone responsive LNCaP prostate cancer cells (Figure 2B). Since LNCaP cells express little WT1 [22], they were transfected with a green fluorescent protein (GFP)-tagged WT1 expression construct 48 hours prior to the ChIP assay. After crosslinking, the chromatin and TF complexes were immunoprecipitated by both WT1 and SP1antibodies, as demonstrated by PCR amplification of the promoter region. WT1 and SP1 may bind at adjacent sites within the *PSA *promoter or at overlapping sites, since the SP1 site overlaps the EGR1 site, to which WT1 may also bind [7-9]. The importance of these WT1 and SP1 TFBS as candidate binding sites was confirmed by the *in vivo *ChIP assay.

### Functional WT1 and SP1 binding sites in the *VEGF *promoter are conserved between human and other primates

**Figure 3:**
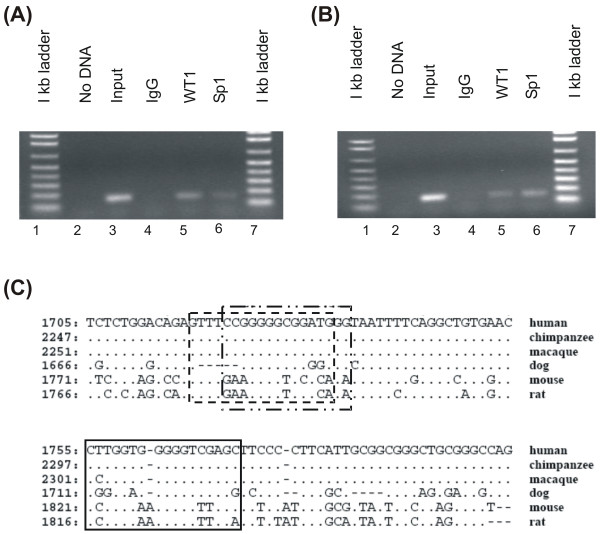
**ChIP verification of WT1 and SP1 binding to endogenous *VEGF *promoter and sequence conservation**. Functional WT1 and SP1 TFBS in the *VEGF *promoter region were previously identified by EMSA and luciferase reporter assays [15]. **(A) **ChIP analysis of chromatin from WT1 transfected 293 kidney cells verified that these TFBS were functional. Lanes 1 and 7 show the 1 Kb ladder, lane 2 shows the No DNA PCR control, and lane 3 shows PCR amplified input DNA. Lanes 4, 5, and 6 show PCR amplified DNA immunoprecipitated by IgG (no antibody control), WT1 or SP1 antibodies, respectively. **(B) **ChIP analysis of chromatin from WT1 transfected LNCaP cells verified these TFBS were functional in prostate cancer cells as well. Lanes as described in section **(A)**. **(C) **Predicted TFBS are based on human sequences and marked by boxes as described in Figure 1. These functional WT1 (human 1755–1771), EGR1 (human 1717–1733) and SP1 (human 1721–1735) sites were conserved between primates (human, chimpanzee, and macaque) and dogs, but not in rodents; and the SP1 site overlapped with the EGR1 site.

Having tested the significance of identified evolutionary conserved sites, we then asked whether TFBS known to mediate transcriptional regulation would also be conserved. Two genes that regulate prostate cancer progression by enhancing growth and blood supply, *AR *and *VEGF*, have multiple WT1 and SP1 binding sites in their proximal promoter regions [14,15,23-25]. We have previously identified an EGR1 site in the *VEGF *promoter that binds both WT1 and SP1 protein *in vitro *[15], and here demonstrate by ChIP assay that this promoter region binds WT1 and SP1 *in vivo *(Figure 3). Chromatin from both embryonic kidney 293 cells and LNCaP cells expressing a GFP-tagged WT1 expression construct was immunoprecipitated by WT1 and SP1 antibodies and amplified by PCR. Using primers specific for the *VEGF *proximal promoter region, products ~140 bp in size were amplified from chromatin of both 293 and LNCaP cells (Figure 3A and 3B). These ChIP assays also demonstrated selective WT1 binding, since an adjacent site 190 nucleotides downstream failed to bind WT1 in the same assay (data not shown). These sites were validated as being transcriptionally regulated in several different assays, including luciferase reporter assays [15], so we asked whether they were evolutionary conserved in different species. *In silico *analyses predicted that an overlapping EGR1 (human 1717–1733) and SP1 (human 1721–1735) site and a WT1 site (human 1755–1771) were conserved between primates and dogs, but not in rodents (Figure 3C). Furthermore, as seen with the *PSA *promoter region, WT1 and SP1 may bind at adjacent sites or potentially at overlapping sites since WT1 also binds at EGR1 sites [7-9]. Both *PSA *and *VEGF *promoter regions contain evolutionary conserved WT1 sites adjacent to overlapping EGR1/SP1 TFBS, to which WT1 is also likely to bind, thus facilitating either cooperation or competition between TFs.

**Figure 4:**
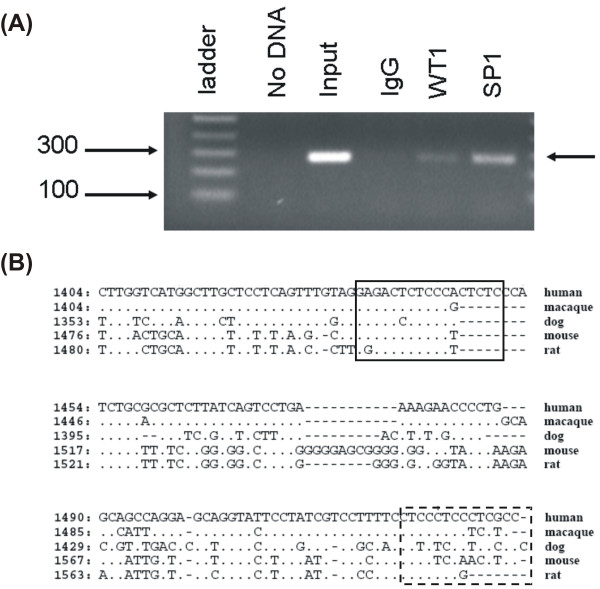
**ChIP verification of WT1 and SP1 binding to endogenous *AR *promoter and sequence analysis**. Functional WT1 TFBS in *AR *promoter region were previously identified by EMSA and reporter assays [14,23]. **(A) **ChIP analysis of chromatin from WT1 transfected LNCaP prostate cancer cells verified these TFBS were functional. Lane 1 shows the 1 Kb ladder, lane 2 shows the No DNA PCR control, and lane 3 shows PCR amplified input DNA. Lanes 4, 5, and 6 show PCR amplified DNA immunoprecipitated by IgG (no antibody control), WT1 or SP1 antibodies, respectively. **(B) **Predicted TFBS are based on human sequences and marked by boxes as described in Figure 1. Evidence for conservation of the functional WT1 (human 1434–1450) TFBS was limited by lack of sequence information available for chimpanzee (and lack of conservation with macaque). Surprisingly the TCC rich EGR1 site (human 1524–1537), previously shown to bind WT1 *in vitro *[14], also showed no evolutionary conservation.

Similarly, WT1 binding sites previously identified in the *AR *proximal promoter region by EMSA analysis and verified to mediate transcriptional regulation in luciferase reporter assays [14,23] were confirmed by ChIP using PCR primers flanking the WT1 and SP1 TFBS (Figure 4A). Since these binding sites were tested *in vivo*, evidence of sequence conservation was sought, as described. As shown in Figure 4B, both a WT1 site (human 1434–1450) and an EGR1 site (human 1524–1537) were identified within the region amplified by ChIP. This less common pyrimidine-rich EGR1 TFBS, consisting of TCC repeats, has been shown to bind both WT1 and SP1 [7,14,26], thus all three zinc finger TFs could compete for binding at this site. Evidence for evolutionary conservation between human and other primates was limited by the lack of genomic sequence information available for chimpanzee (and lack of conservation between human and macaque).

## Discussion

Identification of evolutionary conserved sequences derived from comparisons of multiple genomes (so-called "phylogenetic footprints") has been successful in identifying functionally important regions, including those regions that regulate gene expression [19,27-34]. However, some regulatory genomic sequences do not appear to be conserved or the level of evolutionary conservation varies between different genomic comparisons [35,36]. Importantly, some functional regions have been reported to experience a relatively fast rate of turnover, where the functional significance of the element is retained despite changes at the nucleotide sequence level (e.g., transcription start sites, [37]). Thus, it is likely that gene expression in mammalian genomes is controlled by both types of regulatory elements, i.e., those elements that exhibit evolutionary and functional conservation and those that exhibit functional conservation only. Moreover, while numerous algorithms are available to computationally predict potential regulatory elements, it is often challenging to narrow down the list of those that are likely to be functional, particularly for relatively short elements such as TFBS. One of the approaches that utilizes evolutionary conservation as a predictor of TFBS functionality is the rVISTA tool that uses pairwise sequence alignments to identify the most highly conserved TFBS between the pair of genomic sequences [38]. Another set of tools, the Mulan, takes advantage of evolutionary conservation information obtained from multi-sequence alignments of several genomes [39]. However, the latter requires the TFBS to be shared among all genomes present in the alignment [39] and may potentially miss the lineage-specific regulatory elements that are absent from some subsets of genomes. Therefore, in this work we used TFBS elements shared between some but not necessarily all of the available genomes.

We used evolutionary sequence conservation, as determined by both the multi-species sequence alignments and the *in silico *TFBS predictions, to identify those sites most likely to regulate expression of target genes that influence growth of prostate cancer cells. Regulatory regions with functional importance can be expected to exhibit sequence conservation due to selection. Thus, predicted TFBS that are located in the orthologous positions in multiple genomes are likely to be truly functional. Our identification of evolutionary conserved WT1 and SP1 binding sites in the *PSA *promoter indeed supports this notion (Figure 2). As expected, conservation of TFBS decreased as species became more evolutionarily divergent [40], so those TFBS that were conserved between multiple species including opossum are more likely to be functionally important in the regulation of gene expression.

The abundance of overlapping zinc finger TFBS also supported the functional importance of these regulatory regions. Thus, we identified many TFBS in potential target genes that were co-expressed with WT1 in prostate cancer epithelial cells. Evolutionary conserved WT1 and SP1 sites in the *PSA *promoter were confirmed by ChIP to bind both WT1 and SP1 in LNCaP prostate cancer cells chromatin. Although it is a novel discovery that both SP1 and WT1 bind the *PSA *promoter and may play a role in its regulation, reporter assays are needed to confirm their contribution to transcription. In addition, a WT1 binding site known to transcriptionally regulate the *VEGF *promoter [15] was confirmed by ChIP and found to be in an evolutionary conserved region. Interestingly, transcriptionally active WT1 and EGR1 binding sites in the *AR *promoter [12] were not conserved between human and macaque, although adjacent genomic regions could be aligned between multiple species (Figure 4). This suggests that the *AR *promoter may have experienced faster turn-over than the *VEGF *promoter, yet remained functionally conserved despite sequence changes at the nucleotide level.

Many of the genes expressed in prostate cancer epithelial cells have previously been reported to interact and regulate each other, suggesting multiple potential targets for altered pathways that may lead to prostate cancer progression. We and others have identified gene interactions [8,14,15,23,41-47] that are consistent with WT1 regulating the progression and/or growth of tumors in the prostate. However, *PSA *was a candidate gene target identified by our *in silico *evolutionary conservation approach and confirmed by *in vivo *chromatin binding assays. PSA is a member of the kallikrein family of serine proteases and is a marker of epithelial differentiation in the prostate [48]. It is up-regulated in prostate cancer cells when compared to normal adjacent tissue [49] and its expression is regulated by the ligand bound androgen receptor (AR) [48]. Since WT1 activates the *AR *promoter in prostate cancer cells [23], this suggests that WT1 may directly or indirectly regulate *PSA *gene expression.

In addition to *PSA*, genes that were co-expressed with WT1 in prostate cancer epithelial cells and that could potentially interact with, or be regulated by, WT1 included *GATA2, ECAD, EGR1*, and *NDRG1 *[6]. GATA binding proteins are zinc finger transcription factors that bind the WGATAR consensus motif and are expressed in multiple tissues, including endocrine glands [50-52]. Interestingly, GATA TFs regulate WT1 expression, as multiple GATA TFBS are found within the *WT1 *promoter and enhancer regions [53-55]. GATA binding protein 2 (GATA2) has been shown to be one of the main GATA family members expressed in the prostate of human and mouse [56]. It has been suggested that GATA2 plays a role in androgen mediated regulation of PSA expression, possibly through interaction with AR, as GATA sites are adjacent to AR TFBS in the *PSA *promoter [56]. WT1 could contribute to GATA2 mediated regulation of target genes in prostate cancer cells, if WT1 also physically interacts with GATA2. This notion is consistent with the observation that WT1 interacts with GATA4 to regulate *SRY *gene expression [57]. This complex pattern of zinc finger-protein interaction between WT1 and GATA, along with regulation of WT1 expression by GATA TF, suggests a potential for WT1 feedback control of GATA activity.

The *WT1 *promoter is itself a target of autoregulation by WT1 [47]. WT1 is a multifunctional transcription factor; its four major isoforms are formed by alternative splicing at two sites resulting in the inclusion or exclusion of (1) exon V and/or (2) a tripeptide (KTS) in exon 9 that alters the zinc finger DNA binding structure [58]. While the functions of the various isoforms of WT1 are still being discovered, the -KTS isoform is a transcriptional regulator with G-rich recognition sequence [58]. The +KTS isoform is also likely to be present in prostate cancer cells but would contribute to gene regulation via splicing and post-transcriptional gene regulation [59,60]. Here we have identified potential target genes with well-described DNA binding sites recognized by the -KTS isoform and have not assessed the less well understood RNA binding sites recognized by the +KTS isoform [60].

The early growth response 1 gene (*EGR1*) is a homolog of *WT1 *[7]. Although it has only three zinc-fingers, it shares some TFBS with WT1. *EGR1 *has been implicated as a cancer suppressor gene and activates genes required for differentiation [7]. In human prostate cancer, EGR1 is over-expressed [11,12] and in a mouse model of prostate cancer, EGR1 regulates genes essential for progression of tumor growth [61]. Since WT1 regulates the *EGR1 *promoter *in vitro *[8] it may indirectly regulate other *EGR1 *target genes, such as the N-myc downstream regulated gene 1 (*NDRG1*), an α/β hydrolase. In many cancer cell lines it has been shown to be up-regulated by both hypoxia and hormone treatment suggesting that it could be linked to androgen induced differentiation and signaling in the prostate [62,63]. Since EGR1 regulates NDRG1, WT1 could either directly or indirectly regulate NDRG1.

While analyzing the homologous sequences of the different gene promoters, numerous overlapping TFBS were found, suggesting competition for binding and differential regulation of these gene promoters. Several studies have shown that EGR1 and SP1 TFBS often overlap [7,64,65]. When EGR1 binds to a site also bound by SP1, it displaces the SP1 "activator" from the binding site and represses transcription of these genes [7]. For example, the promoter of *NDRG1 *was shown to be regulated by an overlapping EGR1/SP1 binding site [65] (located outside of the surveyed region of our study). It was shown that this evolutionary conserved site was vital in positively regulating expression of NDRG1 [65]. Similarly, our results showed evolutionary conserved overlapping EGR1/SP1 sites in several other gene promoters, including *VEGF *and *PSA*. In the latter, overlapping EGR1/SP1 sites were found to be conserved between human and two other primate species (chimpanzee and macaque).

Additionally, WT1 and EGR1 compete for binding at shared TFBS. WT1 recognizes and binds to EGR1 sites on the promoters of many different genes [7,9,66-68]. WT1 generally functions as a transcriptional repressor when bound to EGR1 TFBS in the transforming growth factor-beta 1 (*TGFβ1*) and *EGR1 *promoters, while EGR1 functions as an activator [8,9]. Many gene promoters with overlapping WT1, EGR1, and SP1 binding sites have been identified (reviewed in [7]). For example, three-way competition occurs between EGR1, SP1 and WT1 for binding and regulation of superoxide dismutase expression [66]. However, the mechanisms of gene regulation at overlapping sites, including TF competition, are not well understood.

Combinations of adjacent and overlapping EGR1, WT1 and SP1 TFBS conserved between multiple species were found in multiple gene promoters. Adjacent sites were found in the *PSA *promoter where an overlapping EGR1/SP1 site is 50 base pairs downstream of a WT1 site and in the *VEGF *promoter where an EGR1/SP1 overlapping site is 20 base pairs away from a WT1 site. Such sites can facilitate synergistic interactions or may be required for inducible expression, as described for AR and GATA2 interactions in the *PSA *promoter [56]. Additionally, in the *VEGF *promoter an SP1 site adjacent to a non-canonical estrogen receptor (ER) TFBS contributes to hormone induction of VEGF expression [69]. Similarly, WT1 appears to interact with ER at neighboring sites in the insulin like growth factor 1 receptor (*IGF1R*) promoter [70]. These complex arrangements of EGR1, WT1 and SP1 TFBS could facilitate cooperative or competitive binding by these TFs and would have pleiotropic effects on the regulation of these genes. Genes with evolutionary conserved overlapping TFBS could be part of a prostate epithelial cell transcriptome regulated by WT1.

## Conclusion

Genes coordinately expressed in prostate cancer epithelial cells have conserved regulatory elements and an abundance of overlapping zinc finger TFBS. Potential WT1 gene targets were identified based on TFBS sequence conservation, and the significance of the WT1 TFBS in the *PSA *promoter was verified *in vivo *by ChIP assays. Similarly, a transcriptionally active WT1 binding site in the *VEGF *promoter was confirmed by ChIP and found to be in a region conserved amongst primates. Thus, these genes could be part of a novel network of regulatory pathways initiated by WT1 and have important implications in the progression of prostate cancer.

## Methods

### Promoter sequence compilation

For each of the 24 prostate cancer growth regulatory genes differentially expressed, the complete or draft genomes of eight different mammalian species were downloaded from the Ensembl Genome Browser [71,72]. The following genome assemblies were used: the NCBI 36 assembly of human (*Homo sapiens*) genome, the NCBI m36 assembly of mouse (*Mus musculus*) genome, the Pan Tro 2.1 assembly of chimp (*Pan troglodytes*) genome, a whole genome shotgun (WGS) preliminary assembly Btau_3.1 of cow (*Bos Taurus*) genome, a WGS assembly Can Fam2.0 of dog (*Canis familiaris*) genome, a WGS preliminary assembly Mmul_1 of rhesus monkey (*Macaca mulatta*) genome, the Mon Dom5 assembly of opossum (*Monodelphis domestica*) genome, and the RGSC3.4 assembly of rat (*Rattus noregicus*) genome. Since major regulatory elements are located within several hundred base pairs of transcription start sites [73], 1.5 kb of human nucleotide sequence 5' of the translational start site (that is, 5' of the first exon as defined in Ensembl [72]) was collected. Orthologous sequences from other mammalian genomes were obtained from respective genome assemblies. In the case of the *EGR1 *promoter this extended beyond 1.5 kb, so was assigned a negative number. The genome viewer and annotation program Artemis was used to ensure the correct context of genomic sequences [74]. In each sequence, the nucleotide positions were numbered sequentially, with the targeted promoter region occupying positions 1 through 1500 (5' to 3' direction) of the forward strand, and ATG start codon located at positions 1501–1503 of the genomic sequence analyzed.

*AR *and *VEGF *promoter sequences containing the functional WT1 TFBS for the human *AR *and *VEGF *promoters were obtained from Ensembl (ENSG00000169083 and ENSG00000112715, respectively). For alignment analyses of known functional sites [14,15], an orthologous promoter region (3 kb) was then collected from eight mammalian genomes as described above.

### TFBS predictions, evolutionary conservation and multiple sequence alignments

TFBS of WT1, EGR1, SP1, SP2, AP2 and GATA1 were predicted for each gene by the program MatInspector [75] that utilizes the TRANSFAC libraries of TF binding motifs [75,76]. The default parameters of similarity thresholds were used for all examined genes, and they were as follows: core similarity > 0.75 and optimized matrix similarity thresholds (i.e., those that minimize false positives for each individual matrix as available in the library) [75]. In MatInspector, core similarity is one of the built-in program parameters that determines whether the observed sequence match will be analyzed further. It refers to the four most conserved consecutive nucleotides of the matrix, usually the most critical sites for protein binding, and reaches 1.0 only when there is a perfect match [75,77]. Sequence matches with low core similarity (less than 0.75) are not, by default, reported to the user. Vertebrate matrices of the Matrix Family Library Version 6.2 (October 2006) that included 464 matrices were used [78]. Multiple sequence alignments of the promoter sequences were reconstructed with the program blastZ using MultiPipMaker [79], and predicted human TFBS were mapped onto the alignments.

Regions that are conserved in multiple genomes are often found to correspond to functionally important ones [80]. However, because of the species-specific differences in gene regulation due to underlying differences in morphogenesis and development, such as those between different segments of human and rodent prostate [81], it can be expected that some functionally important regions will be conserved only in a limited set of genomes where they play a critical role. Thus, we used a flexible definition of "evolutionary conservation" to accommodate such potential differences between genes and/or TFBS: here a TFBS was considered evolutionary conserved if it was predicted as a respective TFBS in orthologous position in at least three of eight surveyed genomes. In other words, the same genomic region was predicted to function as a candidate binding site for a particular TF in at least 3 surveyed genomes. Further, because differences in presence/absence of particular TFB sites between genomes may also be attributed to differences in the role of respective genes in each of the organisms, we examined evolutionary conserved sites at different levels of resolution: Human-Primates, Human-Rodents, and Human-Opossum, thereby, allowing us to identify genes and TFB sites that are functionally relevant to each of these comparisons.

### Cell culture and chromatin immunoprecipitation

LNCaP prostate cancer cells (ATCC-CRL 1740) and human embryonic kidney 293 cells (ATCC-CRL 1573) were cultured in RPMI or DEM/F12 (HyClone Laboratories, Utah) media, respectively, as described [15]. The cytomegalovirus (CMV) promoter-driven pGFP-WT1 (A) expression construct encoding the murine *Wt1 *gene (lacking both KTS insertion and exon 5) fused to GFP coding region were obtained from Dr. A. Ward [82]. All DNA was purified by the Qiagen plasmid Maxi Kit (Qiagen, Carlsbad CA) and transfections were performed using lipofectamine 2000 (Invitrogen; Carlsbad CA) in serum- and antibiotic-free media as described [15]. Green fluorescing cells were visualized by epifluorescence microscopy (Olympic) at 100–400× magnification at 24 and 48 hrs after transfection prior to cell harvest for chromatin isolation.

The Farnham ChIP protocol [83] was used with some modifications. Two million cells were treated with formaldehyde to crosslink proteins to DNA and lysed in PBS-PI as recommended for the EZ ChIP Assay (Upstate Biotechnology Inc). Lysates were centrifuged and DNA sheared by sonication (Biosonik III, Bronwill Scientific, Rochester, NY) to fragments of 100–400 bp in length. The supernatant was pre-cleared by incubation with Protein G Agarose and incubated overnight at 4°C with either SP1 antibody (Upstate Biotechnology Inc) or WT1 antibody (a mixture of C19 and N18 polyclonal Abs, Santa Cruz Biotechnology) or non-immune IgG. The antibody/protein/DNA complex was collected by incubation with Protein G Agarose and washed in increasing salt buffers, then rinsed in TE as recommended (Upstate Biotechnology Inc). The complexes were recovered from agarose beads with an elution buffer, crosslinks were reversed and DNA was purified using G-50 spin columns. Four percent of both immunoprecipitated and input chromatin were amplified by PCR using *Taq *polymerase (Applied Biosystems by Roche Molecular System, Inc) and the following set of primers: *VEGF *primers (F) 5'TTCCTAGCAAAGAGGGAACG3' and (R) 5'ACCAAGGTTCACAGCCTGAA3'; *AR *primers (F) 5'TATCTGCTGGCTTGGTCATGGCTTG3' and (R) 5'CTGCTTCCTGAATAGCTCCTGCTT3'; and *PSA *primers (F) 5'TCTGCCTTTGTCCCCTAGAT3' and (R) 5'AACCTTCATTCCCCAGGACT3'. Following an initial 10 min denaturation at 95°C, DNA was amplified by 32 cycles of: 1) 20 sec denaturation at 95°C, 2) 30 sec annealing at either 58°C (for *VEGF *primers) or 59°C (for *AR *and *PSA *primers) and 3) 30 sec extension at 72°C; amplification was completed with a 2 min final extension at 72°C. PCR products were fractionated on 1% agarose gel, and ethidium bromide stained DNA was visualized by a gel doc system (BIORAD, CA). Specificity controls are shown in Additional file 2.

## Abbreviations

TFs: transcription factors; *WT1*: Wilms tumor 1; TFBS: transcription factor binding sites; ChIP: chromatin immunoprecipitation; *AR*: androgen receptor; *VEGF*: vascular endothelial growth factor; *PSA*: prostate specific antigen; *KLK3*: kallikrein-related peptidase 3; PCR: polymerase chain reaction; GFP: green fluorescent protein.

## Authors' contributions

KE performed ChIP analysis, *in silico *analysis, and drafted the text. ST performed *in silico *analysis. ABazarov performed *in silico *analysis. ABrett performed ChIP analysis and contributed to the text. GF planned functional assays, guided student research, and drafted the text. HP planned *in silico *analyses, guided student research, and drafted the text.

## Supplementary Material

Additional file 1 Evolutionary conserved TFBS in promoters of 24 genes expressed in prostate cancer epithelium. This table lists evolutionary conserved transcription factor binding sites in promoters of 24 genes expressed in prostate cancer. Click here for file

Additional file 2 WT1 bound the proximal, but not the distal, region of the amphiregulin (AREG) gene promoter in chromatin of LNCaP cells. This specificity control illustrates WT1 binding to the proximal region (known to bind WT1), but not the distal region of the AREG promoter in LNCaP chromatin. Click here for file

## References

[B1] Jemal A, Siegel R, Ward E, Murray T, Xu J, Thun MJ (2007). Cancer statistics, 2007. CA Cancer J Clin.

[B2] Stamey TA, Caldwell M, McNeal JE, Nolley R, Hemenez M, Downs J (2004). The prostate specific antigen era in the United States is over for prostate cancer: what happened in the last 20 years?. J Urol.

[B3] Luo JH, Yu YP, Cieply K, Lin F, Deflavia P, Dhir R, Finkelstein S, Michalopoulos G, Becich M (2002). Gene expression analysis of prostate cancers. Mol Carcinog.

[B4] Singh D, Febbo PG, Ross K, Jackson DG, Manola J, Ladd C, Tamayo P, Renshaw AA, D'Amico AV, Richie JP, Lander ES, Loda M, Kantoff PW, Golub TR, Sellers WR (2002). Gene expression correlates of clinical prostate cancer behavior. Cancer Cell.

[B5] Chandran UR, Dhir R, Ma C, Michalopoulos G, Becich M, Gilbertson J (2005). Differences in gene expression in prostate cancer, normal appearing prostate tissue adjacent to cancer and prostate tissue from cancer free organ donors. BMC Cancer.

[B6] Brown K (2006). Differential Gene Expression Patterns in Prostate Cancer Epithelial and Interstitial Stromal Cells.

[B7] Liu C, Calogero A, Ragona G, Adamson E, Mercola D (1996). EGR-1, the reluctant suppression factor: EGR-1 is known to function in the regulation of growth, differentiation, and also has significant tumor suppressor activity and a mechanism involving the induction of TGF-beta1 is postulated to account for this suppressor activity. Crit Rev Oncog.

[B8] Madden SL, Cook DM, Morris JF, Gashler A, Sukhatme VP, Rauscher FJ (1991). Transcriptional repression mediated by the WT1 Wilms tumor gene product. Science.

[B9] Dey BR, Sukhatme VP, Roberts AB, Sporn MB, Rauscher FJ, Kim SJ (1994). Repression of the transforming growth factor-beta 1 gene by the Wilms' tumor suppressor WT1 gene product. Mol Endocrinol.

[B10] Devilard E, Bladou F, Ramuz O, Karsenty G, Dales JP, Gravis G, Nguyen C, Bertucci F, Xerri L, Birnbaum D (2006). FGFR1 and WT1 are markers of human prostate cancer progression. BMC Cancer.

[B11] Ogishima T, Shiina H, Breault JE, Tabatabai L, Bassett WW, Enokida H, Li LC, Kawakami T, Urakami S, Ribeiro-Filho LA, Terashima M, Fujime M, Igawa M, Dahiya R (2005). Increased heparanase expression is caused by promoter hypomethylation and up-regulation of transcriptional factor early growth response-1 in human prostate cancer. Clin Cancer Res.

[B12] Eid MA, Kumar MV, Iczkowski KA, Bostwick DG, Tindall DJ (1998). Expression of early growth response genes in human prostate cancer. Cancer Res.

[B13] Rivera MN, Haber DA (2005). Wilms' tumour: connecting tumorigenesis and organ development in the kidney. Nat Rev Cancer.

[B14] Shimamura R, Fraizer GC, Trapman J, Lau Y, Saunders GF (1997). The Wilms' tumor gene WT1 can regulate genes involved in sex determination and differentiation: SRY, Mullerian-inhibiting substance, and the androgen receptor. Clin Cancer Res.

[B15] Hanson J, Gorman J, Reese J, Fraizer G (2007). Regulation of vascular endothelial growth factor, VEGF, gene promoter by the tumor suppressor, WT1. Front Biosci.

[B16] Thomas JW, Touchman JW (2002). Vertebrate genome sequencing: building a backbone for comparative genomics. Trends in Genetics.

[B17] Dubchak I, Brudno M, Loots GG, Pachter L, Mayor C, Rubin EM, Frazer KA (2000). Active conservation of noncoding sequences revealed by three-way species comparisons. Genome Res.

[B18] Stojanovic N, Florea L, Riemer C, Gumucio D, Slightom J, Goodman M, Miller W, Hardison R, Journals O (1999). Comparison of five methods for finding conserved sequences in multiple alignments of gene regulatory regions. Nucleic Acids Res.

[B19] Wang H, Zhang Y, Cheng Y, Zhou Y, King DC, Taylor J, Chiaromonte F, Kasturi J, Petrykowska H, Gibb B, Dorman C, Miller W, Dore LC, Welch J, Weiss MJ, Hardison RC (2006). Experimental validation of predicted mammalian erythroid cis-regulatory modules. Genome Res.

[B20] Johnson DS, Davidson B, Brown CD, Smith WC, Sidow A (2004). Noncoding regulatory sequences of Ciona exhibit strong correspondence between evolutionary constraint and functional importance. Genome Res.

[B21] Hedges S, Kumar S (2003). Genomic clocks and evolutionary timescales. Trends in Genetics.

[B22] Fraizer G, Leahy R, Priyadarshini S, Graham K, Delacerda J, Diaz M (2004). Suppression of prostate tumor cell growth *in vivo* by WT1, the Wilms' tumor suppressor gene. Int J Oncol.

[B23] Kohler B, Delezoide AL, Boizet-Bonhoure B, McPhaul MJ, Sultan C, Lumbroso S (2007). Coexpression of Wilms' tumor suppressor 1 (WT1) and androgen receptor (AR) in the genital tract of human male embryos and regulation of AR promoter activity by WT1. J Mol Endocrinol.

[B24] Husbeck B, Bhattacharyya RS, Feldman D, Knox SJ (2006). Inhibition of androgen receptor signaling by selenite and methylseleninic acid in prostate cancer cells: two distinct mechanisms of action. Mol Cancer Ther.

[B25] Pore N, Gupta AK, Cerniglia GJ, Jiang Z, Bernhard EJ, Evans SM, Koch CJ, Hahn SM, Maity A (2006). Nelfinavir down-regulates hypoxia-inducible factor 1alpha and VEGF expression and increases tumor oxygenation: implications for radiotherapy. Cancer Res.

[B26] Englert C, Hou X, Maheswaran S, Bennett P, Ngwu C, Re GG, Garvin AJ, Rosner MR, Haber DA (1995). WT1 suppresses synthesis of the epidermal growth factor receptor and induces apoptosis. EMBO J.

[B27] Tagle DA, Koop BF, Goodman M, Slightom JL, Hess DL, Jones RT (1988). Embryonic and globin genes of a prosimian primate (Galago crassicaudatus): Nucleotide and amino acid sequences, developmental regulation and phylogenetic footprints. J Mol Biol.

[B28] Elnitski L, Miller W, Hardison R (1997). Conserved E boxes function as part of the enhancer in hypersensitive site 2 of the beta-globin locus control region. Role of basic helix-loop-helix proteins. J Biol Chem.

[B29] Hardison RC, Oeltjen J, Miller W (1997). Long human-mouse sequence alignments reveal novel regulatory elements: a reason to sequence the mouse genome. Genome Res.

[B30] Vuillaumier S, Dixmeras I, Messai H, Lapoumeroulie C, Lallemand D, Gekas J, Chehab FF, Perret C, Elion J, Denamur E (1997). Cross-species characterization of the promoter region of the cystic fibrosis transmembrane conductance regulator gene reveals multiple levels of regulation. Biochem J.

[B31] Loots GG, Locksley RM, Blankespoor CM, Wang ZE, Miller W, Rubin EM, Frazer KA (2000). Identification of a coordinate regulator of interleukins 4, 13, and 5 by cross-species sequence comparisons. Science.

[B32] Cliften P, Sudarsanam P, Desikan A, Fulton L, Fulton B, Majors J, Waterston R, Cohen BA, Johnston M (2003). Finding functional features in *saccharomyces* genomes by phylogenetic footprinting. Science.

[B33] Kellis M, Patterson N, Endrizzi M, Birren B, Lander ES (2003). Sequencing and comparison of yeast species to identify genes and regulatory elements. Nature.

[B34] Xie X, Lu J, Kulbokas EJ, Golub TR, Mootha V, Lindblad-Toh K, Lander ES, Kellis M (2005). Systematic discovery of regulatory motifs in human promoters and 3' UTRs by comparison of several mammals. Nature.

[B35] Hughes JR, Cheng JF, Ventress N, Prabhakar S, Clark K, Anguita E, De Gobbi M, de Jong P, Rubin E, Higgs DR (2005). Annotation of cis-regulatory elements by identification, subclassification, and functional assessment of multispecies conserved sequences. Proc Natl Acad Sci USA.

[B36] King DC, Taylor J, Elnitski L, Chiaromonte F, Miller W, Hardison RC (2005). Evaluation of regulatory potential and conservation scores for detecting cis-regulatory modules in aligned mammalian genome sequences. Genome Res.

[B37] Frith MC, Ponjavic J, Fredman D, Kai C, Kawai J, Carninci P, Hayshizaki Y, Sandelin A (2006). Evolutionary turnover of mammalian transcription start sites. Genome Res.

[B38] Loots GG, Ovcharenko I (2004). rVISTA 2.0: evolutionary analysis of transcription factor binding sites. Nucleic Acids Res.

[B39] Ovcharenko I, Loots GG, Giardine BM, Hou M, Ma J, Hardison RC, Stubbs L, Miller W (2005). Mulan: Multiple-sequence local alignment and visualization for studying function and evolution. Genome Res.

[B40] Dermitzakis ET, Clark AG (2002). Evolution of transcription factor binding sites in mammalian gene regulatory regions: conservation and turnover. Mol Biol Evol.

[B41] Hewitt SM, Hamada S, McDonnell TJ, Rauscher FJ, Saunders GF (1995). Regulation of the proto-oncogenes bcl-2 and c-myc by the Wilms' tumor suppressor gene WT1. Cancer Res.

[B42] Mayo MW, Wang CY, Drouin SS, Madrid LV, Marshall AF, Reed JC, Weissman BE, Baldwin AS (1999). WT1 modulates apoptosis by transcriptionally upregulating the bcl-2 proto-oncogene. EMBO J.

[B43] Werner H, Rauscher FJ, Sukhatme VP, Drummond IA, Roberts CT, LeRoith D (1994). Transcriptional repression of the insulin-like growth factor I receptor (IGF-I-R) gene by the tumor suppressor WT1 involves binding to sequences both upstream and downstream of the IGF-I-R gene transcription start site. J Biol Chem.

[B44] Werner H, Re GG, Drummond IA, Sukhatme VP, Rauscher FJ, Sens DA, Garvin AJ, LeRoith D, Roberts CT (1993). Increased expression of the insulin-like growth factor I receptor gene, IGF1R, in Wilms tumor is correlated with modulation of IGF1R promoter activity by the WT1 Wilms tumor gene product. Proc Natl Acad Sci U S A.

[B45] Drummond IA, Madden SL, Rohwer-Nutter P, Bell GI, Sukhatme VP, Rauscher FJ (1992). Repression of the insulin-like growth factor II gene by the Wilms tumor suppressor WT1. Science.

[B46] Hosono S, Gross I, English MA, Hajra KM, Fearon ER, Licht JD (2000). E-cadherin is a WT1 target gene. J Biol Chem.

[B47] Hewitt SM, Fraizer GC, Wu YJ, Rauscher FJ, Saunders GF (1996). Differential function of Wilms' tumor gene WT1 splice isoforms in transcriptional regulation. J Biol Chem.

[B48] Yin H, Radomska HS, Tenen DG, Glass J (2006). Down regulation of PSA by C/EBPalpha is associated with loss of AR expression and inhibition of PSA promoter activity in the LNCaP cell line. BMC Cancer.

[B49] Borgono CA, Diamandis EP (2004). The emerging roles of human tissue kallikreins in cancer. Nat Rev Cancer.

[B50] Merika M, Orkin SH (1993). DNA-binding specificity of GATA family transcription factors. Mol Cell Biol.

[B51] Tremblay JJ, Viger RS (2003). Novel roles for GATA transcription factors in the regulation of steroidogenesis. J Steroid Biochem Mol Biol.

[B52] Viger RS, Guittot SM, Anttonen M, Wilson DB, Heikinheimo M (2008). Role of the GATA family of transcription factors in endocrine development, function, and disease. Mol Endocrinol.

[B53] Fraizer GC, Wu YJ, Hewitt SM, Maity T, Ton CC, Huff V, Saunders GF (1994). Transcriptional regulation of the human Wilms' tumor gene (WT1). cell type-specific enhancer and promiscuous promoter. J Biol Chem.

[B54] Wu Y, Fraizer GC, Saunders GF (1995). GATA-1 Transactivates the WT1 Hematopoietic Specific Enhancer. J Biol Chem.

[B55] Zhang X, Xing G, Fraizer GC, Saunders GF (1997). Transactivation of an Intronic Hematopoietic-specific Enhancer of the Human Wilms' Tumor 1 Gene by GATA-1 and c-Myb. J Biol Chem.

[B56] Perez-Stable CM, Pozas A, Roos BA (2000). A role for GATA transcription factors in the androgen regulation of the prostate-specific antigen gene enhancer. Mol Cell Endocrinol.

[B57] Miyamoto Y, Silversides D, Viger R GATA4 Enhances SRY Gene Transcription Through a Direct Interaction with Wilms Tumor 1 (WT1). Biology of Reproduction 2005.

[B58] Rauscher FJ, Morris JF, Tournay OE, Cook DM, Curran T (1990). Binding of the Wilms' tumor locus zinc finger protein to the EGR-1 consensus sequence. Science.

[B59] Larsson SH, Charlieu JP, Miyagawa K, Engelkamp D, Rassoulzadegan M, Ross A, Cuzin F, van Heyningen V, Hastie ND (1995). Subnuclear localization of WT1 in splicing or transcription factor domains is regulated by alternative splicing. Cell.

[B60] Ladomery M, Sommerville J, Woolner S, Slight J, Hastie N (2003). Expression in Xenopus oocytes shows that WT1 binds transcripts *in vivo*, with a central role for zinc finger one. J Cell Sci.

[B61] Abdulkadir SA, Qu Z, Garabedian E, Song SK, Peters TJ, Svaren J, Carbone JM, Naughton CK, Catalona WJ, Ackerman JJ, Gordon JI, Humphrey PA, Milbrandt J (2001). Impaired prostate tumorigenesis in Egr1-deficient mice. Nat Med.

[B62] Ellen T, Ke Q, Zhang P, Costa M (2008). NDRG1, a Growth and Cancer Related Gene: Regulation of Gene Expression and Function in Normal and Disease States. Carcinogenesis.

[B63] Caruso RP, Levinson B, Melamed J, Wieczorek R, Taneja S, Polsky D, Chang C, Zeleniuch-Jacquotte A, Salnikow K, Yee H, Costa M, Osman I (2004). Altered N-myc downstream-regulated gene 1 protein expression in African-American compared with caucasian prostate cancer patients. Clin Cancer Res.

[B64] Rong Y, Hu F, Huang R, Mackman N, Horowitz JM, Jensen RL, Durden DL, Van Meir EG, Brat DJ (2006). Early growth response gene-1 regulates hypoxia-induced expression of tissue factor in glioblastoma multiforme through hypoxia-inducible factor-1-independent mechanisms. Cancer Res.

[B65] Zhang P, Tchou-Wong KM, Costa M (2007). Egr-1 Mediates Hypoxia-Inducible Transcription of the NDRG1 Gene through an Overlapping Egr-1/Sp1 Binding Site in the Promoter. Cancer Res.

[B66] Minc E, de Coppet P, Masson P, Thiery L, Dutertre S, Amor-Gueret M, Jaulin C (1999). The human copper-zinc superoxide dismutase gene (SOD1) proximal promoter is regulated by Sp1, Egr-1, and WT1 via non-canonical binding sites. J Biol Chem.

[B67] Harrington MA, Konicek B, Song A, Xia XL, Fredericks WJ, Rauscher FJ (1993). Inhibition of colony-stimulating factor-1 promoter activity by the product of the Wilms' tumor locus. J Biol Chem.

[B68] Wang ZY, Madden SL, Deuel TF, Rauscher FJ (1992). The Wilms' tumor gene product, WT1, represses transcription of the platelet-derived growth factor A-chain gene. J Biol Chem.

[B69] Stoner M, Wormke M, Saville B, Samudio I, Qin C, Abdelrahim M, Safe S (2004). Estrogen regulation of vascular endothelial growth factor gene expression in ZR-75 breast cancer cells through interaction of estrogen receptor alpha and SP proteins. Oncogene.

[B70] Reizner N, Maor S, Sarfstein R, Abramovitch S, Welshons WV, Curran EM, Lee AV, Werner H (2005). The WT1 Wilms' tumor suppressor gene product interacts with estrogen receptor-alpha and regulates IGF-I receptor gene transcription in breast cancer cells. J Mol Endocrinol.

[B71] Ensembl Genome Browser. http://www.ensembl.org/.

[B72] Hubbard TJ, Aken BL, Beal K, Ballester B, Caccamo M, Chen Y, Clarke L, Coates G, Cunningham F, Cutts T, Down T, Dyer SC, Fitzgerald S, Fernandez-Banet J, Graf S, Haider S, Hammond M, Herrero J, Holland R, Howe K, Howe K, Johnson N, Kahari A, Keefe D, Kokocinski F, Kulesha E, Lawson D, Longden I, Melsopp C, Megy K, Meidl P, Ouverdin B, Parker A, Prlic A, Rice S, Rios D, Schuster M, Sealy I, Severin J, Slater G, Smedley D, Spudich G, Trevanion S, Vilella A, Vogel J, White S, Wood M, Cox T, Curwen V, Durbin R, Fernandez-Suarez XM, Flicek P, Kasprzyk A, Proctor G, Searle S, Smith J, Ureta-Vidal A, Birney E (2007). Ensembl 2007. Nucleic Acids Res.

[B73] Dieterich C, Cusack B, Wang H, Rateitschak K, Krause A, Vingron M (2002). Annotating regulatory DNA based on man-mouse genomic comparison. Bioinformatics.

[B74] Rutherford K, Parkhill J, Crook J, Horsnell T, Rice P, Rajandream MA, Barrell B (2000). Artemis: sequence visualization and annotation. Bioinformatics.

[B75] Cartharius K, Frech K, Grote K, Klocke B, Haltmeier M, Klingenhoff A, Frisch M, Bayerlein M, Werner T (2005). MatInspector and beyond: promoter analysis based on transcription factor binding sites. Bioinformatics.

[B76] Wingender E, Chen X, Hehl R, Karas H, Liebich I, Matys V, Meinhardt T, Prüß M, Reuter I, Schacherer F (2000). TRANSFAC: an integrated system for gene expression regulation. Nucleic Acids Res.

[B77] Werner T (2000). Computer-assisted analysis of transcription control regions. Methods Mol Biol.

[B78] Genomatix-understanding gene regulation. http://www.genomatix.de.

[B79] Schwartz S, Elnitski L, Li M, Weirauch M, Riemer C, Smit A, Green ED, Hardison RC, Miller W, NISC Comparative Sequencing Program (2003). MultiPipMaker and supporting tools: alignments and analysis of multiple genomic DNA sequences. Nucleic Acids Res.

[B80] Frazer KA, Tao H, Osoegawa K, de Jong PJ, Chen X, Doherty MF, Cox DR (2004). Noncoding Sequences Conserved in a Limited Number of Mammals in the SIM2 Interval are Frequently Functional. Genome Res.

[B81] Berquin IM, Min Y, Wu R, Wu H, Chen YQ (2005). Expression signature of the mouse prostate. J Biol Chem.

[B82] Dutton JR, Lahiri D, Ward A (2006). Different isoforms of the Wilms' tumour protein WT1 have distinct patterns of distribution and trafficking within the nucleus. Cell Prolif.

[B83] Weinmann AS, Farnham PJ (2002). Identification of unknown target genes of human transcription factors using chromatin immunoprecipitation. Methods.

[B84] Hanson J, Brown K, Reese J, Gorman J, Cash J, Graham K, Fraizer GC (2006). Wilms' tumor suppressor gene, WT1, modulates VEGF expression and is differentially expressed in prostate tumor cells. Proc Amer Assoc Cancer Res.

[B85] Sroka IC, Nagle RB, Bowden GT (2007). Membrane-type 1 matrix metalloproteinase is regulated by sp1 through the differential activation of AKT, JNK, and ERK pathways in human prostate tumor cells. Neoplasia.

[B86] Phan D, Cheng CJ, Galfione M, Vakar-Lopez F, Tunstead J, Thompson NE, Burgess RR, Najjar SM, Yu-Lee LY, Lin SH (2004). Identification of Sp2 as a Transcriptional Repressor of Carcinoembryonic Antigen-Related Cell Adhesion Molecule 1 in Tumorigenesis. Cancer Res.

[B87] Ho SM, Leung YK, Chung I (2006). Estrogens and antiestrogens as etiological factors and therapeutics for prostate cancer. Ann N Y Acad Sci.

[B88] Ruiz M, Pettaway C, Song R, Stoeltzing O, Ellis L, Bar-Eli M (2004). Activator protein 2alpha inhibits tumorigenicity and represses vascular endothelial growth factor transcription in prostate cancer cells. Cancer Res.

[B89] Zhang X, Leung YK, Ho SM (2007). AP-2 regulates the transcription of estrogen receptor (ER)-beta by acting through a methylation hotspot of the oN promoter in prostate cancer cells. Oncogene.

[B90] Rhodes DR, Sanda MG, Otte AP, Chinnaiyan AM, Rubin MA (2003). Multiplex biomarker approach for determining risk of prostate-specific antigen-defined recurrence of prostate cancer. J Natl Cancer Inst.

[B91] Mehrian-Shai R, Chen CD, Shi T, Horvath S, Nelson SF, Reichardt JK, Sawyers CL (2007). Insulin growth factor-binding protein 2 is a candidate biomarker for PTEN status and PI3K/Akt pathway activation in glioblastoma and prostate cancer. Proc Natl Acad Sci USA.

[B92] Chatterjee S, Park ES, Soloff MS (2004). Proliferation of DU145 prostate cancer cells is inhibited by suppressing insulin-like growth factor binding protein-2. Int J Urol.

[B93] Le H, Arnold JT, McFann KK, Blackman MR (2006). DHT and testosterone, but not DHEA or E2, differentially modulate IGF-I, IGFBP-2, and IGFBP-3 in human prostatic stromal cells. Am J Physiol Endocrinol Metab.

[B94] Nagpal ML, Davis J, Lin T (2006). Overexpression of CXCL10 in human prostate LNCaP cells activates its receptor (CXCR3) expression and inhibits cell proliferation. Biochim Biophys Acta.

[B95] Tu LC, Yan X, Hood L, Lin B (2007). Proteomics analysis of the interactome of N-myc downstream regulated gene 1 and its interactions with the androgen response program in prostate cancer cells. Mol Cell Proteomics.

[B96] Kim KH, Dobi A, Shaheduzzaman S, Gao CL, Masuda K, Li H, Drukier A, Gu Y, Srikantan V, Rhim JS, Srivastava S (2007). Characterization of the androgen receptor in a benign prostate tissue-derived human prostate epithelial cell line: RC-165N/human telomerase reverse transcriptase. Prostate Cancer Prostatic Dis.

[B97] Ruscica M, Dozio E, Boghossian S, Bovo G, Martos Riano V, Motta M, Magni P (2006). Activation of the Y1 receptor by neuropeptide Y regulates the growth of prostate cancer cells. Endocrinology.

[B98] Rasiah KK, Kench JG, Gardiner-Garden M, Biankin AV, Golovsky D, Brenner PC, Kooner R, O'neill GF, Turner JJ, Delprado W, Lee CS, Brown DA, Breit SN, Grygiel JJ, Horvath LG, Stricker PD, Sutherland RL, Henshall SM (2006). Aberrant neuropeptide Y and macrophage inhibitory cytokine-1 expression are early events in prostate cancer development and are associated with poor prognosis. Cancer Epidemiol Biomarkers Prev.

[B99] Liu P, Ramachandran S, Ali Seyed M, Scharer CD, Laycock N, Dalton WB, Williams H, Karanam S, Datta MW, Jaye DL, Moreno CS (2006). Sex-determining region Y box 4 is a transforming oncogene in human prostate cancer cells. Cancer Res.

[B100] Vanaja DK, Ballman KV, Morlan BW, Cheville JC, Neumann RM, Lieber MM, Tindall DJ, Young CY (2006). PDLIM4 repression by hypermethylation as a potential biomarker for prostate cancer. Clin Cancer Res.

[B101] Wang H, McKnight NC, Zhang T, Lu ML, Balk SP, Yuan X (2007). SOX9 is expressed in normal prostate basal cells and regulates androgen receptor expression in prostate cancer cells. Cancer Res.

[B102] Drivdahl R, Haugk KH, Sprenger CC, Nelson PS, Tennant MK, Plymate SR (2004). Suppression of growth and tumorigenicity in the prostate tumor cell line M12 by overexpression of the transcription factor SOX9. Oncogene.

[B103] ExPASy-UniProt Knowledgebase: Swiss-Prot and TrEMBL. http://ca.expasy.org/sprot/.

[B104] Ashburner M, Ball CA, Blake JA, Botstein D, Butler H, Cherry JM, Davis AP, Dolinski K, Dwight SS, Eppig JT, Harris MA, Hill DP, Issel-Tarver L, Kasarskis A, Lewis S, Matese JC, Richardson JE, Ringwald M, Rubin GM, Sherlock G (2000). Gene ontology: tool for the unification of biology. The Gene Ontology Consortium. Nat Genet.

[B105] Dennis G, Sherman BT, Hosack DA, Yang J, Gao W, Lane HC, Lempicki RA (2003). DAVID: Database for Annotation, Visualization, and Integrated Discovery. Genome Biol.

