# Supplementary material for: Evolutionary conservation of zinc finger transcription factor binding sites in promoters of genes co-expressed with WT1 in prostate cancer
Source: BMC Genomics. 2008 Jul 16;9:337. doi: 10.1186/1471-2164-9-337 (PMC2515153; doi:10.1186/1471-2164-9-337)
Supplement: Additional file 1 — Evolutionary conserved TFBS in promoters of 24 genes expressed in prostate cancer epithelium. This table lists evolutionary conserved transcription factor binding sites in promoters of 24 genes expressed in prostate cancer. [file 1471-2164-9-337-S1.pdf]

Additional files

Additional file 1- Evolutionary conserved TFBS in promoters of 24 genes expressed in prostate cancer epithelium.

Gene	Conserved between[a]	WT1		EGR1		SP1		SP2		AP2		GATA1	
		Conserved sites[b]	Total # of predicted sites[c]	Conserved sites[b]	Total # of predicted sites[c]	Conserved sites[b]	Total # of predicted sites[c]	Conserved sites[b]	Total # of predicted sites[c]	Conserved sites[b]	Total # of predicted sites[c]	Conserved sites[b]	Total # of predicted sites[c]
**ABCC4**	**H-Pr**	1/1	1	10/4	11	1/1	4	1/1	1	2/2	2	4/3	5
	**H-Ro**	PA/*		1/*		1/*		0/*		0/*		0/*	
	**H-Op**	*		*		*		*		*		*	
**ACPP**	**H-Pr**	NP/NP	NP	NP/NP	NP	NP/NP	NP	NP/NP	NP	NP/NP	NP	2/2	3
	**H-Ro**	NP/NP		NP/NP		NP/NP		NP/NP		NP/NP		PA/PA	
	**H-Op**	*		*		*		*		*		*	
**AMD1**	**H-Pr**	1/PA	1	1/PA	1	1/PA	1	1/PA	1	1/PA	1	3/PA	4
	**H-Ro**	PA/0		PA/0		PA/0		PA/0		PA/0		PA/0	
	**H-Op**	0		0		0		0		0		0	
**CLDN8**	**H-Pr**	NP/NP	NP	NP/NP	NP	NP/NP	NP	NP/NP	NP	NP/NP	NP	3/3	4
	**H-Ro**	NP/NP		NP/NP		NP/NP		NP/NP		NP/NP		0/0	
	**H-Op**	NP		NP		NP		NP		NP		PA	
**ECAD**	**H-Pr**	1/PA	2	1/0	1	3/0	4	1/0	2	1/PA	1	NP/NP	0
	**H-Ro**	0/0		PA/PA		0/0		0/0		0/0		NP/NP	
	**H-Op**	*		*		*		*		*		*	
**EGR1**	**H-Pr**	4/4	8	7/8	10	3/4	7	1/0	1	1/1	1	3/1	3
	**H-Ro**	2/2		3/4		2/2		0/0		0/0		1/0	
	**H-Op**	1		2		1		0		PA		0	

**FGFR3**	**H-Pr**	NSA/2	6	NSA/1	7	NSA/3	11	NSA/1	2	NSA/1	4	NSA/NP	NP
	**H-Ro**	0/0		0/0		0/0		0/0		0/0		NP/NP	
	**H-Op**	*		*		*		*		*		*	
**GATA2**	**H-Pr**	7/8	8	1/1	1	3/4	5	3/2	3	2/3	3	NP/NP	NP
	**H-Ro**	1/2		PA/PA		1/0		0/0		0/0		NP/NP	
	**H-Op**	1		PA		PA		PA		0		NP	
**GOLPH2**	**H-Pr**	NP/NP	NP	NP/NP	NP	1/1	1	NP/NP	NP	NP/NP	NP	5/5	5
	**H-Ro**	*/*		*/*		*/*		*/*		*/*		*/*	
	**H-Op**	*		*		*		*		*		*	
**GREB1**	**H-Pr**	1/*	1	1/*	1	1/*	2	1/*	1	1/*	1	2/*	3
	**H-Ro**	*/*		*/*		*/*		*/*		*/*		*/*	
	**H-Op**	*		*		*		*		*		*	
**IGFBP2**	**H-Pr**	3/2	3	6/6	6	6/3	7	1/1	1	NP/NP	NP	3/1	3
	**H-Ro**	0/0		3/1		1/1		0/0		NP/NP		0/0	
	**H-Op**	0		1		1		0		NP		0	
**KLK3**	**H-Pr**	2/2	3	1/1	1	1/1	2	1/0	1	NP/NP	NP	1/1	2
	**H-Ro**	NSA/NSA		NSA/NSA		NSA/NSA		NSA/NSA		NSA/NSA		NSA/NSA	
	**H-Op**	NSA		NSA		NSA		NSA		NSA		NSA	
**KRT18**	**H-Pr**	2/PA	2	1/PA	1	3/0	6	1/PA	1	NP/NP	NP	1/PA	1
	**H-Ro**	*/*		*/*		*/*		*/*		*/*		*/*	
	**H-Op**	NSA		NSA		NSA		NSA		NSA		NSA	
**NDRG1**	**H-Pr**	1/1	1	NP/NP	NP	NP/NP	NP	NP/NP	NP	0/1	1	1/1	1
	**H-Ro**	0/0		NP/NP		NP/NP		NP /NP		0/0		0/0	
	**H-Op**	*		*		*		*		*		*	
**NPY**	**H-Pr**	9/7	9	2/1	2	4/4	4	2/2	2	4/2	5	2/2	2
	**H-Ro**	1/1		0/0		1/1		1/0		0/0		0/0	
	**H-Op**	0		0		0		PA		1		PA	
**NKX3-1**	**H-Pr**	NP/NP	NP	1/1	1	2/1	2	NP/NP	NP	NP/NP	NP	1/1	2
	**H-Ro**	*/*		NP/0		*/*		*/*		*/*		*/*	
	**H-Op**	NSA		NSA		NSA		NSA		NSA		NSA	
**PSCA**	**H-Pr**	1/0	1	NP/NP	NP	3/1	4	2/1	2	0/0	1	2/2	3
	**H-Ro**	*/*		*/*		*/*		*/*		*/*		*/*	

	**H-Op**	NSA		NSA		NSA		NSA		NSA		NSA	
**PSMA**	**H-Pr**	NP/NP	NP	0/0	1	PA/0	3	NP/NP	NP	NP/NP	NP	2/0	2
	**H-Ro**	*/*		*/*		*/*		*/*		*/*		*/*	
	**H-Op**	*		*		*		*		*		*	
**SHBG**	**H-Pr**	1/PA	1	2/0	2	1/PA	2	3/0	3	3/PA	3	3/PA	4
	**H-Ro**	*/PA		*/PA		*/PA		*/PA		*/PA		*/PA	
	**H-Op**	PA		PA		0		PA		PA		PA	
**SOX4**	**H-Pr**	2/1	2	2/2	2	3/3	3	1/1	1	NP/NP	NP	2/2	2
	**H-Ro**	1/1		0/0		2/2		0/0		NP/NP		2/2	
	**H-Op**	0		0		0		0		NP		0	
**SOX9**	**H-Pr**	3/2	4	1/1	1	4/4	4	NP/NP	NP	NP/NP	NP	NP/NP	NP
	**H-Ro**	0/1		0/PA		1/PA		NP/NP		NP/NP		NP/NP	
	**H-Op**	0		0		0		NP		NP		NP	
**TACSTD1**	**H-Pr**	0/0	1	0/0	3	0/0	4	NP/NP	NP	0/0	1	0/0	1
	**H-Ro**	*/*		*/*		*/*		*/*		*/*		*/*	
	**H-Op**	*		*		*		*		*		*	
**TFAP2C**	**H-Pr**	NP/NP	NP	4/4	4	5/4	8	NP/NP	NP	1/1	1	NP/NP	NP
	**H-Ro**	NP/NP		3/3		2/2		NP/NP		1/1		NP/NP	
	**H-Op**	NP		1		2		NP		PA		NP	
**WT1**	**H-Pr**	4/2	7	3/3	3	3/3	6	1/1	1	2/1	4	1/0	5
	**H-Ro**	1/1		0/0		2/2		0/0		1/0		0/0	
	**H-Op**	1		0		2		0		PA		0	

[a] H-Pr = TFBS conserved between human and other primates (chimpanzee / macaque)

H-Ro = TFBS conserved between human and rodents (mouse / rat)

H-Op = TFBS conserved between human and opossum

[b] PA= only partial alignment of promoters as constructed by MultiPipMaker [72], * = promoter sequences too divergent to align, NP = no TFBS in human promoters as predicted by MatInspector [71], 0 = TFBS not conserved, and NSA = no orthologous sequence is available in Ensembl.

[c] Total number of predicted sites is based on TFBS in human promoters.
