# Supplementary material for: Evolutionary conservation of zinc finger transcription factor binding sites in promoters of genes co-expressed with WT1 in prostate cancer
Source: BMC Genomics. 2008 Jul 16;9:337. doi: 10.1186/1471-2164-9-337 (PMC2515153; doi:10.1186/1471-2164-9-337)
Supplement: Additional file 2 — WT1 bound the proximal, but not the distal, region of the amphiregulin (AREG) gene promoter in chromatin of LNCaP cells. This specificity control illustrates WT1 binding to the proximal region (known to bind WT1), but not the distal region of the AREG promoter in LNCaP chromatin. [file 1471-2164-9-337-S2.pdf]

Additional file 2.

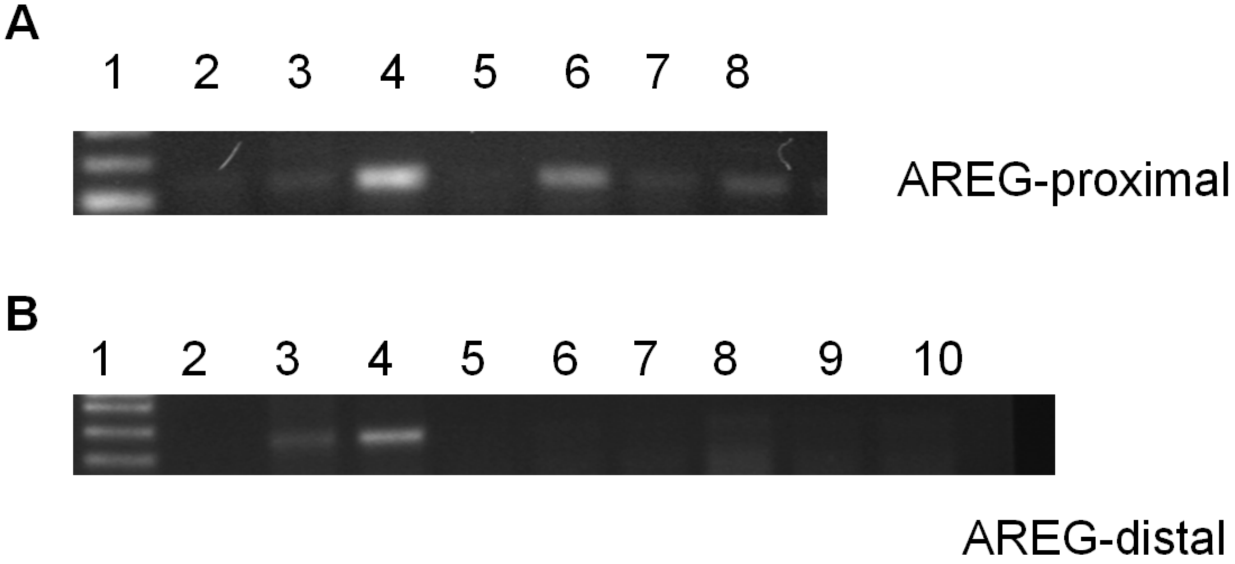

Additional file 2. WT1 bound the proximal, but not the distal, region of the amphiregulin (AREG) gene promoter in chromatin of LNCaP cells. The AREG gene promoter served as a specificity control for chromatin and Ab described in text. The proximal promoter region, known to bind WT1 (Kim et al 2007), served as a positive control, while the distal region was a negative control. PCR primer sequences were kindly proved by Dr. Lee (Kim et al. 2007). **Panel A. WT1 bound the proximal region of the AREG promoter in LNCaP chromatin.** ChIP and PCR amplification were performed as described in text, with the following modifications: PCR buffer contained 5% DMSO and conditions were: 95ºC for 5 min., 33 cycles of (94ºC for 30 sec., 58ºC for 30 sec., and 72ºC for 45 sec.) and then a final extension at 72ºC for 7 min. Lane 1: 100 bp molecular weight marker. Lane 2: No DNA PCR control. Lanes 3 and 4: Input DNA (diluted 1:5 and undiluted, respectively). Lane 5: Anti-IgG antibody pull-down (Upstate). Lane 6: Anti-WT1 antibody pull-down (rabbit monoclonal Ab, Epitomics). Lanes 7 and 8: Anti-WT1 antibody pull-down (C19/N18, Santa Cruz). Input represents 4% of total starting chromatin and monoclonal Ab had not previously been tested. **Panel B. WT1 did not bind the distal region of the AREG promoter in LNCaP chromatin.** ChIP and PCR analysis were performed as described for panel A, except 30 cycles of PCR were used. Lane 1: 100 bp molecular weight marker. Lane 2: No DNA PCR control. Lanes 3 and 4 : Input DNA (diluted 1:5 and undiluted, respectively). Lanes 5 and 6: Anti-IgG antibody pull-down (diluted 1:5 and undiluted, respectively). Lanes 7 and 8: Anti-WT1 (C19, Santa Cruz) antibody pull-down (diluted 1:5 and undiluted, respectively). Lanes 9 and 10: Anti-WT1 (C19/N18, Santa Cruz) antibody pull-down (diluted 1:5 and undiluted, respectively). Input represents 4% of total starting chromatin and PCR amplified products were undetectable in the WT1 pull-down fractions.

Reference:

Kim, H. S., M. S. Kim, A. L. Hancock, J. C. Harper, J. Y. Park, G. Poy, A. O. Perantoni, M. Cam, K. Malik, and S. B. Lee. 2007. Identification of novel Wilms' tumor suppressor gene target genes implicated in kidney development. J Biol Chem **282**:16278-16287.
